# Microbiota and metabolomic profiling coupled with machine learning to identify biomarkers and drug targets in nasopharyngeal carcinoma

**DOI:** 10.3389/fphar.2025.1551411

**Published:** 2025-02-26

**Authors:** Junsong Liu, Chongwen Xu, Rui Wang, Jianhua Huang, Ruimin Zhao, Rui Wang

**Affiliations:** ^1^ Department of Otorhinolaryngology-Head and Neck Surgery, The First Affiliated Hospital of Xi’an Jiaotong University, Xi’an, Shaanxi, China; ^2^ Department of Thoracic Surgery, The First Affiliated Hospital of Xi’an Jiaotong University, Cancer Centre, Xi’an, Shaanxi, China; ^3^ Department of Anesthesiology, The First Affiliated Hospital of Xi’an Jiaotong University, Xi’an, Shaanxi, China

**Keywords:** nasopharyngeal carcinoma, radiotherapy resistance, microbiota, *Bacteroides* acidifaciens, acetate, short-chain fatty acids, XGBoost, biomarkers

## Abstract

**Background:**

Nasopharyngeal carcinoma (NPC) is a prevalent malignancy in certain regions, with radiotherapy as the standard treatment. However, resistance to radiotherapy remains a critical challenge, necessitating the identification of novel biomarkers and therapeutic targets. The tumor-associated microbiota and metabolites have emerged as potential modulators of radiotherapy outcomes.

**Methods:**

This study included 22 NPC patients stratified into radiotherapy-responsive (R, n = 12) and radiotherapy-non-responsive (NR, n = 10) groups. Tumor tissue and fecal samples were subjected to 16S rRNA sequencing to profile microbiota composition and targeted metabolomics to quantify short-chain fatty acids (SCFAs). The XGBoost algorithm was applied to identify microbial taxa associated with radiotherapy response, and quantitative PCR (qPCR) was used to validate key findings. Statistical analyses were conducted to assess differences in microbial diversity, relative abundance, and metabolite levels between the groups.

**Results:**

Significant differences in alpha diversity at the species level were observed between the R and NR groups. *Bacteroides acidifaciens* was enriched in the NR group, while *Propionibacterium acnes* and *Clostridium magna* were more abundant in the R group. Machine learning identified *Acidosoma*, *Propionibacterium acnes*, and *Clostridium magna* as key predictors of radiotherapy response. Metabolomic profiling revealed elevated acetate levels in the NR group, implicating its role in tumor growth and immune evasion. Validation via qPCR confirmed the differential abundance of these microbial taxa in both tumor tissue and fecal samples.

**Discussion:**

Our findings highlight the interplay between microbiota and metabolite profiles in influencing radiotherapy outcomes in NPC. These results suggest that targeting the microbiota-metabolite axis may enhance radiotherapy efficacy in NPC.

## Introduction

Nasopharyngeal carcinoma (NPC) is a malignancy originating from the epithelial cells of the nasopharynx, distinguished by its unique geographic and ethnic distribution, with particularly high incidences in Southeast Asia and Southern China (Su et al., 2024; Zhang et al., 2023). Globally, NPC accounts for more than 130,000 new cases and over 80,000 deaths annually, with most cases occurring in endemic regions such as Guangdong Province in China (Lei et al., 2024; Yue et al., 2021). Although radiotherapy, often combined with chemotherapy, remains the cornerstone of NPC treatment, a significant subset of patients (approximately 20%–30%) exhibit radiotherapy resistance, resulting in poor clinical outcomes and highlighting the urgent need for novel predictive markers and therapeutic targets to enhance treatment efficacy (Liu et al., 2024; Yip et al., 2024).

Recent evidence has underscored the importance of the tumor microenvironment (TME) and host-associated microbial communities in influencing cancer progression and response to therapy (Kiousi et al., 2023; Saadh et al., 2024). The human microbiota, notably the gut microbiota, exerts profound effects on systemic immunity, modulating both innate and adaptive immune responses and thereby shaping the efficacy of various cancer treatments, including radiotherapy (Cullin et al., 2021; Mattiola and Diefenbach, 2023). Dysbiosis—an imbalance in microbial community composition—has been implicated in tumorigenesis and therapy resistance, with certain microbial taxa promoting a more robust antitumor immune response, while others facilitate an immunosuppressive environment that undermines therapeutic interventions (Li et al., 2022; Rajasekaran et al., 2024).

In parallel, metabolomics, the comprehensive profiling of small-molecule metabolites within biological systems, has emerged as a powerful platform for elucidating the biochemical underpinnings of cancer progression and treatment response (Schmidt et al., 2021). Perturbations in key metabolic pathways—encompassing short-chain fatty acids (SCFAs), amino acid derivatives, and polyamines—have been closely linked to NPC pathobiology and radiotherapy outcomes (Lim et al., 2024). SCFAs, such as acetate, propionate, and butyrate, have been demonstrated to modulate immune responses and strengthen epithelial integrity, potentially enhancing radiosensitivity within the tumor milieu (Thapa et al., 2024).

Technological advances in microbial profiling (e.g., 16S rRNA gene sequencing) and metabolomic analyses (e.g., targeted and untargeted LC-MS/MS approaches) have begun to delineate the complex interplay between microbial ecosystems, metabolic networks, and the TME in NPC(Hazrati et al., 2022; Mishra et al., 2021). However, the extraction of meaningful insights from these large, multidimensional datasets remains challenging. To address this, machine learning (ML) and other computational approaches have been increasingly employed to identify predictive microbial and metabolic signatures associated with therapeutic response, offering the prospect of more precise stratification and personalized interventions (Ghannam and Techtmann, 2021).

In this study, we leveraged integrated microbiota and metabolomic profiling of tumor tissue and fecal samples derived from NPC patients stratified into radiotherapy-responsive (R) and radiotherapy-resistant (NR) cohorts. By applying advanced ML-based analytics to these complementary datasets, we aimed to uncover novel biomarkers and metabolic pathways that predict radiotherapy resistance and to identify putative drug targets for improving clinical outcomes in NPC.

## Methods

### Patient recruitment and sample collection

This study aimed to investigate the microbial and metabolomic characteristics associated with radiotherapy response in nasopharyngeal carcinoma (NPC) patients. A total of 22 NPC patients were recruited, including 14 males (63.6%) and 8 females (36.4%), with a median age of 45 years (range: 30–67). The clinical staging according to the eighth edition of the UICC/AJCC system included 2 patients (9.1%) with stage I, 5 (22.7%) with stage II, 10 (45.5%) with stage III, and 5 (22.7%) with stage IV. Tumor tissue and fecal samples were collected prior to the initiation of radiotherapy. Based on treatment efficacy evaluated by radiological and clinical assessments, patients were classified into radiotherapy-responsive (R, n = 12) and radiotherapy-non-responsive (NR, n = 10) groups (Supplementary Table S1). The study was conducted in accordance with the Declaration of Helsinki and approved by the Xi’an Jiaotong University Ethics Committee. Written informed consent was obtained from all participants. Tumor tissue biopsies were obtained during routine diagnostic procedures, and fecal samples were collected using sterile fecal collection kits (Thermo Fisher Scientific, Waltham, MA, United States). All samples were immediately snap-frozen in liquid nitrogen and stored at −80°C until analysis.

### DNA extraction and 16S rRNA gene sequencing

For DNA extraction, approximately 200 mg of tumor tissue or fecal matter was processed using the QIAamp DNA Mini Kit (Qiagen, Hilden, Germany) following the manufacturer’s protocol. Lysis was enhanced with proteinase K (Qiagen) at 56°C for 30 min, followed by spin-column purification. DNA purity and concentration were measured using a NanoDrop spectrophotometer (Thermo Fisher Scientific) and quantified with a Qubit 4 fluorometer (Thermo Fisher Scientific). Extracted DNA was stored at −20°C until downstream analysis.

Microbial profiling was performed via 16S rRNA gene sequencing targeting the V3-V4 hypervariable regions. Amplification was conducted using primers 338F (5′-ACT​CCT​ACG​GGA​GGC​AGC​AG-3′) and 806R (5′-GGACTACHVGGGTWTCTAAT-3′). Polymerase chain reaction (PCR) was carried out in 25 μL reactions containing 12.5 μL of 2× KAPA HiFi HotStart ReadyMix (Roche, Basel, Switzerland), 0.2 μM of each primer, and 10 ng of template DNA. Thermal cycling conditions were as follows: initial denaturation at 95°C for 3 min, followed by 25 cycles of denaturation at 95°C for 30 s, annealing at 55°C for 30 s, and extension at 72°C for 30 s, with a final elongation at 72°C for 5 min. Amplicons were purified using AMPure XP beads (Beckman Coulter, Brea, CA, United States) and sequenced on an Illumina MiSeq platform (Illumina, San Diego, CA, United States) with 2 × 250 bp paired end reads. Sequencing data were processed using QIIME2 (v2021.4), and operational taxonomic units (OTUs) were clustered at 97% similarity against the SILVA database (release 138).

### Metabolomic profiling

Targeted metabolomic profiling of short-chain fatty acids (SCFAs) was conducted using gas chromatography-mass spectrometry (GC-MS). Tumor tissues and fecal samples (∼100 mg) were homogenized in 1 mL of ice-cold methanol (HPLC-grade, Merck, Darmstadt, Germany) containing 10 μM of internal standard (2-ethylbutyric acid, Sigma-Aldrich, St. Louis, MO, United States). Samples were centrifuged at 14,000 × g for 10 min at 4°C, and the supernatants were derivatized with N,O-bis(trimethylsilyl)trifluoroacetamide (BSTFA, Sigma-Aldrich). Derivatized samples were injected into an Agilent 7890 GC system coupled to a 5977B MS detector (Agilent Technologies, Santa Clara, CA, United States) using an HP-5MS column (30 m × 0.25 mm × 0.25 μm). Quantification was performed based on calibration curves generated from SCFA standards (Sigma-Aldrich), and results were normalized to sample weight.

### Machine learning analysis

Machine learning analysis was employed to identify microbial taxa associated with radiotherapy response. The XGBoost algorithm was selected due to its ability to handle small to moderate-sized datasets effectively, manage missing values, and provide interpretable feature importance rankings, making it particularly suitable for the objectives of this study. The implementation was performed using the “xgboost” package in R (v4.1.0). Genus- and species-level relative abundance data were input into the model. To optimize the model’s performance, hyperparameters such as learning rate (0.1), maximum tree depth (6), and the number of trees (500) were fine-tuned using a grid search approach with five-fold cross-validation. The feature importance scores, calculated based on the contribution of each feature to reducing the model’s error, were used to rank microbial taxa according to their predictive power. The top three taxa at each taxonomic level were selected for further validation. Additionally, model evaluation metrics, including accuracy, precision, and area under the receiver operating characteristic (ROC) curve, were reported to ensure the robustness of the model.

### Quantitative PCR validation

Quantitative PCR (qPCR) validation was performed for the top microbial taxa identified by XGBoost. Primers were designed using Primer-BLAST (NCBI) to target *Bacteroides acidifaciens*, *Propionibacterium acnes*, and *Clostridium magna* (sequences and primers listed in Supplementary Table S2). qPCR reactions were conducted in 20 μL volumes containing 10 μL of SYBR Green PCR Master Mix (Applied Biosystems, Foster City, CA, United States), 0.4 μM of each primer, and 20 ng of template DNA. Reactions were run on a QuantStudio 5 Real-Time PCR System (Applied Biosystems) under standard cycling conditions. Relative abundance was determined using the ΔΔCt method and normalized to the universal 16S rRNA gene.

### Statistical analysis

Statistical analyses were performed using R software (v4.1.0). Differences in microbial diversity indices, relative abundance, and metabolite concentrations between the R and NR groups were assessed using the Wilcoxon rank-sum test. Adjustments for multiple comparisons were made using the Benjamini–Hochberg procedure, with a significance threshold set at an adjusted p-value of <0.05.

## Results

### Microbiota profiling of tumor tissues reveals differences in alpha diversity and relative abundance between radiotherapy response groups

**FIGURE 1:**
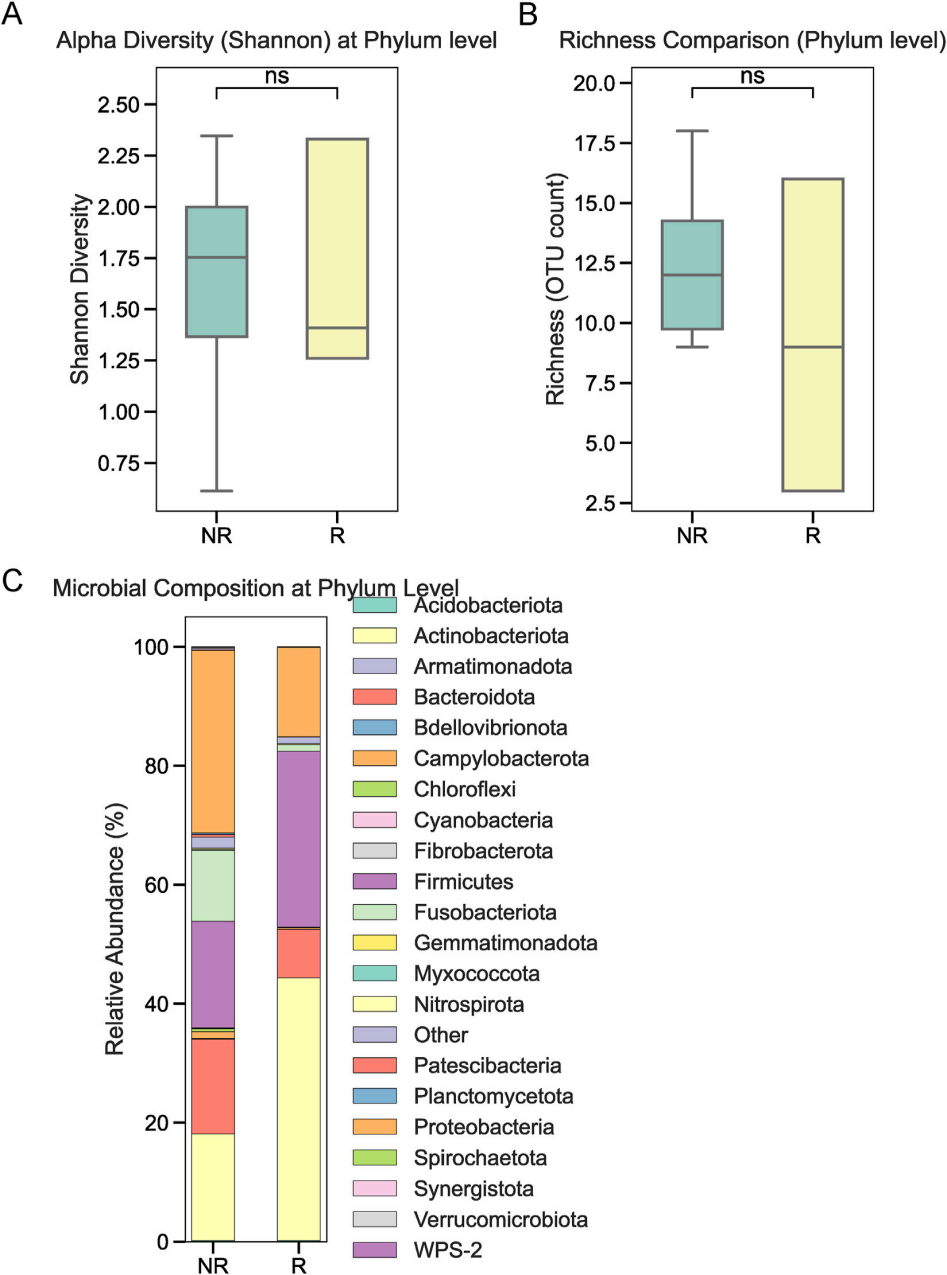
Microbial diversity and relative abundance at the phylum level. **(A)** Alpha diversity (Shannon index) at the phylum level showed no significant differences between the R and NR groups. **(B)** Relative abundance analysis at the phylum level revealed no significant differences between the two groups. **(C)** Proportions of dominant phyla, including Firmicutes and Bacteroidetes, in the R and NR groups.

**FIGURE 2:**
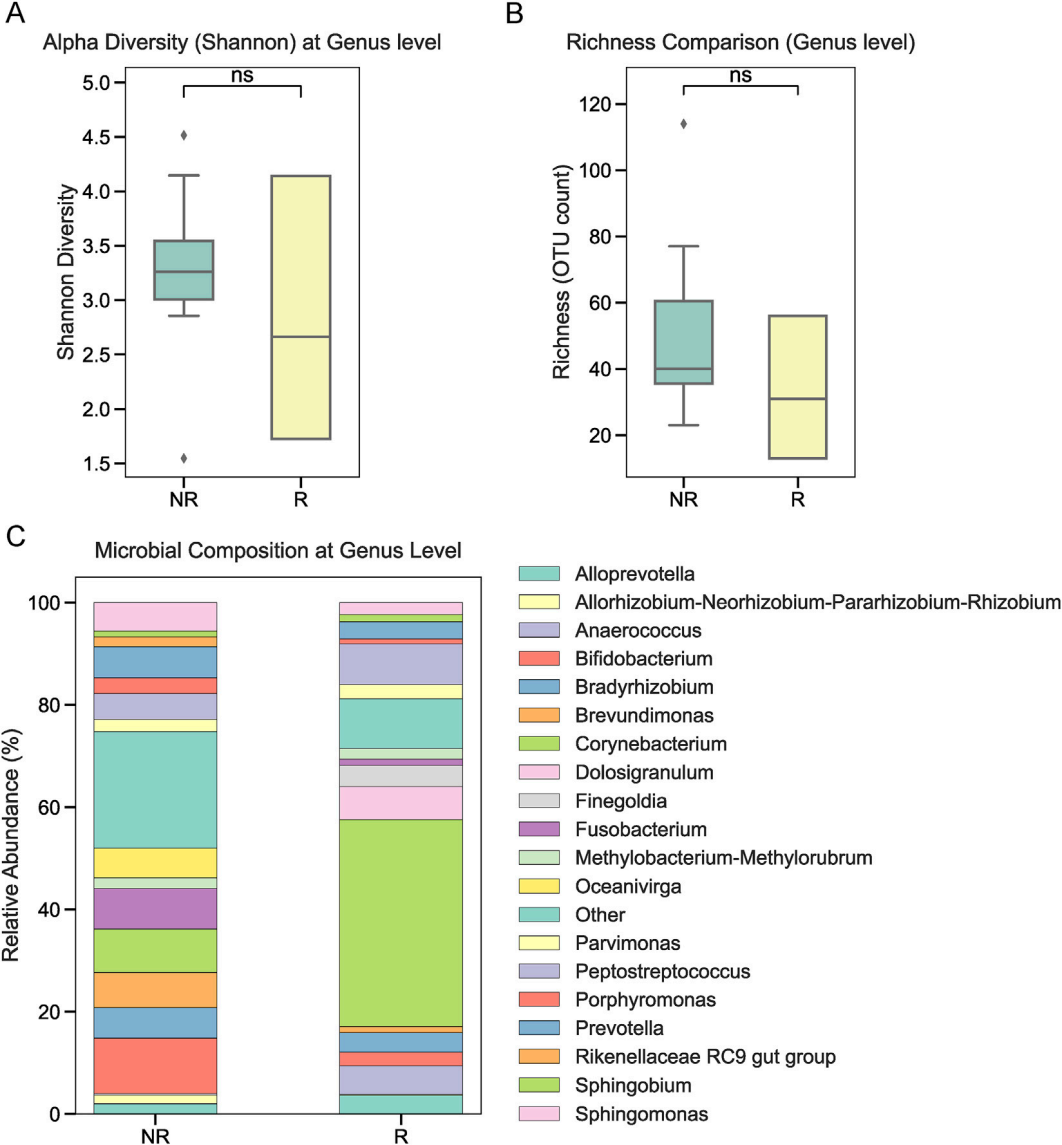
Microbial composition at the genus level. **(A, B)** Alpha diversity and richness at the genus level showed no significant differences between the R and NR groups. **(C)** Relative abundance at the genus level showing consistent microbial composition between the two groups, with prominent genera including *Alloprevotella*, *Fusobacterium*, and *Prevotella*.

**FIGURE 3:**
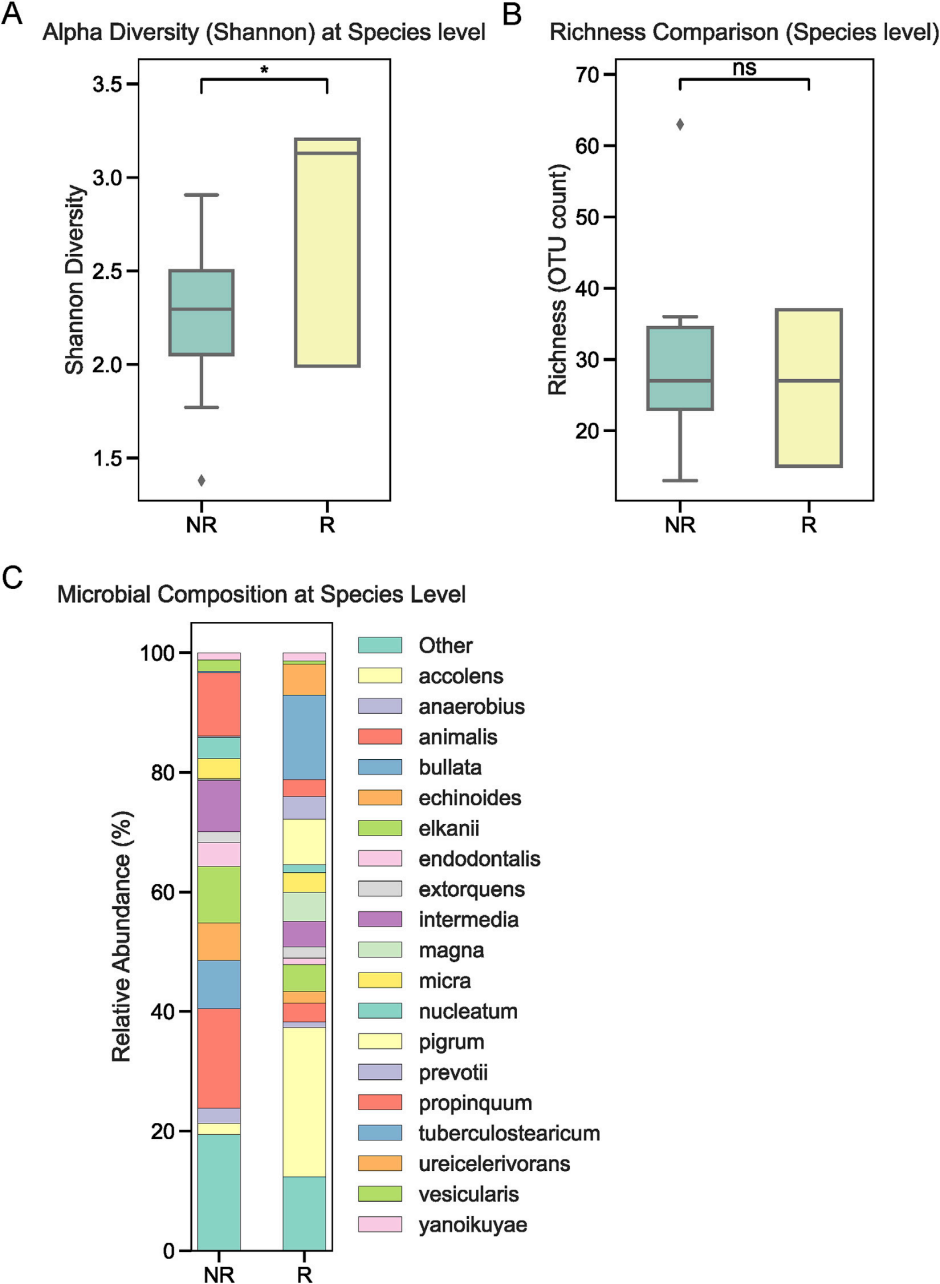
Microbial diversity and composition at the species level. **(A)** Alpha diversity (Shannon index) at the species level was significantly higher in the R group compared to the NR group. **(B)** Richness (OTU counts) showed no significant differences between the groups. **(C)** Relative abundance at the species level revealed taxa such as *Bacteroides acidifaciens* and *Anaerobius* were enriched in the R group, while *Clostridium magna* was more abundant in the NR group.

To investigate the role of tumor-associated microbiota in radiotherapy outcomes, we performed 16S rRNA sequencing on tumor tissues collected from 22 NPC patients prior to treatment. Patients were stratified into radiotherapy-responsive (R) and radiotherapy-non-responsive (NR) groups based on post-treatment efficacy. Alpha diversity analysis at the phylum level showed no significant differences between the two groups (Figure 1A). Similarly, overall relative abundance at the phylum level did not differ significantly between R and NR patients (Figure 1B). However, a closer examination of specific phyla revealed that Firmicutes and Bacteroidetes were the dominant taxa in both groups, with slight variations in their proportions (Figure 1C). At the genus level, relative abundance analysis showed no significant differences in the microbial composition between the R and NR groups (Figures 2A,B). At the genus level, relative abundance analysis showed a consistent microbial composition between the R and NR groups. Genera such as *Alloprevotella*, *Fusobacterium*, and *Prevotella* were among the most prominent taxa in both groups, with slight variations in their relative proportions (Figure 2C). These genera were dominant contributors to the overall microbiota composition, but no significant differences in abundance were observed between the two groups. At the species level, differences in alpha diversity and relative abundance were observed between the R and NR groups. Alpha diversity, measured by the Shannon index, was significantly higher in the R group compared to the NR group (Figure 3A). However, richness, as indicated by OTU counts, did not differ significantly between the two groups (Figure 3B). Relative abundance analysis revealed notable differences in species composition, with taxa such as *Bacteroides acidifaciens* and *Anaerobius* showing higher abundance in the R group, while *Clostridium magna* was enriched in the NR group (Figure 3C).

### Identification of key microbial taxa associated with radiotherapy response using XGBoost

**FIGURE 4:**
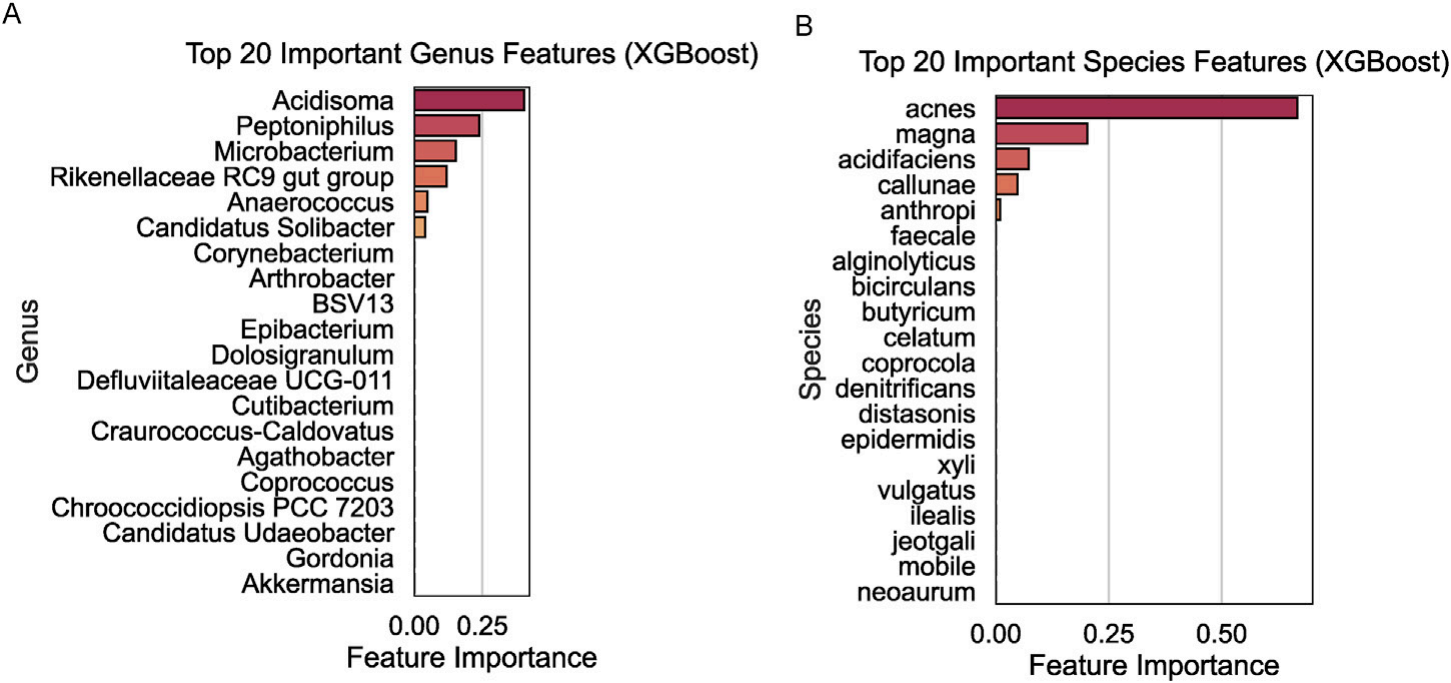
Key microbial taxa identified using XGBoost. **(A)** Top 20 important genus features with *Acidosoma*, *Peptoniphilus*, and *Microbacterium* identified as the top contributors. **(B)** Top 20 important species features with *Propionibacterium acnes*, *Clostridium magna*, and *Bacteroides acidifaciens* as the most discriminative taxa.

To pinpoint microbial taxa with the highest discriminatory power between the R and NR groups, we applied the XGBoost algorithm to genus- and species-level data. At the genus level, *Acidosoma*, *Peptoniphilus*, and *Microbacterium* were identified as the top three contributors to the classification model, with significant feature importance scores (Figure 4A). At the species level, *Propionibacterium acnes*, *Clostridium magna*, and *Bacteroides acidifaciens* emerged as the top three key discriminators between the two groups (Figure 4B).

### Differential abundance of key microbial taxa in tumor tissues and fecal samples

**FIGURE 5:**
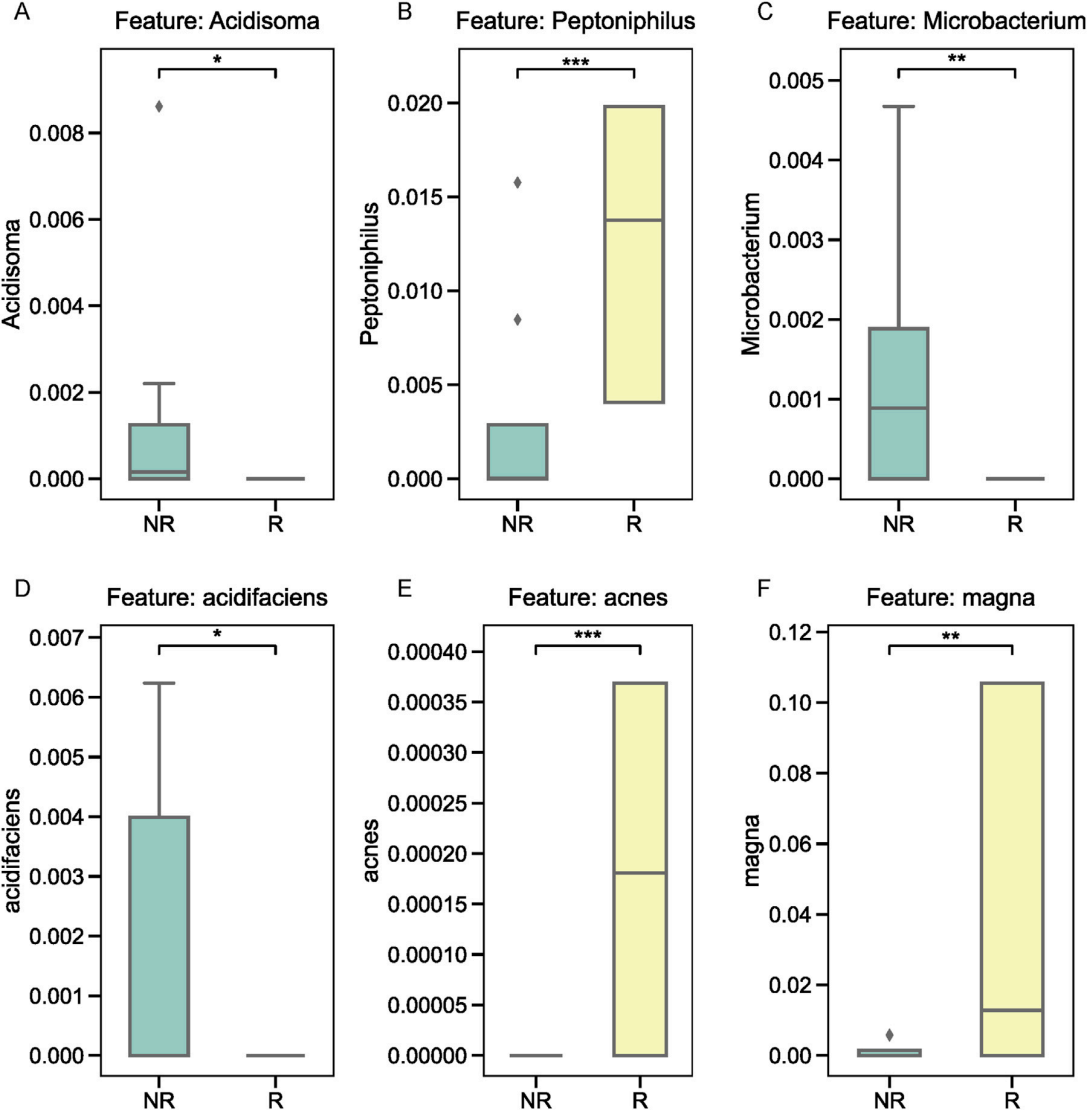
Relative abundance of key microbial taxa in tumor tissues. **(A–C)** Genus-level analysis of *Acidosoma*, *Peptoniphilus*, and *Microbacterium*, showing higher abundance in the R group. **(D–F)** Species-level analysis of *Bacteroides acidifaciens*, *Propionibacterium acnes*, and *Clostridium magna*, with distinct abundance patterns between the R and NR groups.

**FIGURE 6:**
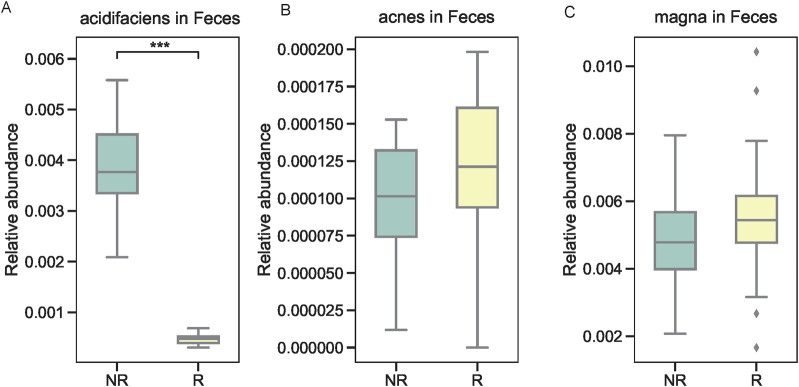
Validation of microbial taxa in fecal samples using qPCR. **(A)** Higher levels of *Bacteroides acidifaciens* in the NR group. **(B, C)** Elevated levels of *Propionibacterium acnes* and *Clostridium magna* in the R group.

We further examined the relative abundance of the top three microbial taxa identified by XGBoost in the R and NR groups. At the genus level, *Acidosoma*, *Peptoniphilus*, and *Microbacterium* were significantly more abundant in the R group compared to the NR group, as shown in Figures 5A–C. At the species level, *Bacteroides acidifaciens*, *Propionibacterium acnes*, and *Clostridium magna* exhibited distinct abundance patterns between the two groups. Specifically, *Bacteroides acidifaciens* was enriched in the NR group (Figure 5D), while *Propionibacterium acnes* and *Clostridium magna* were more abundant in the R group (Figures 5E,F). To validate these findings, we performed quantitative PCR (qPCR) on fecal samples from the same patients, focusing on the three species of interest. The results confirmed the differential abundance of these taxa, with *Bacteroides acidifaciens* showing higher levels in fecal samples from the NR group, whereas *Propionibacterium acnes* and *Clostridium magna* exhibited elevated levels in the R group (Figures 6A–C).

### Short-chain fatty acid profiling highlights acetate as a differential metabolite

**FIGURE 7:**
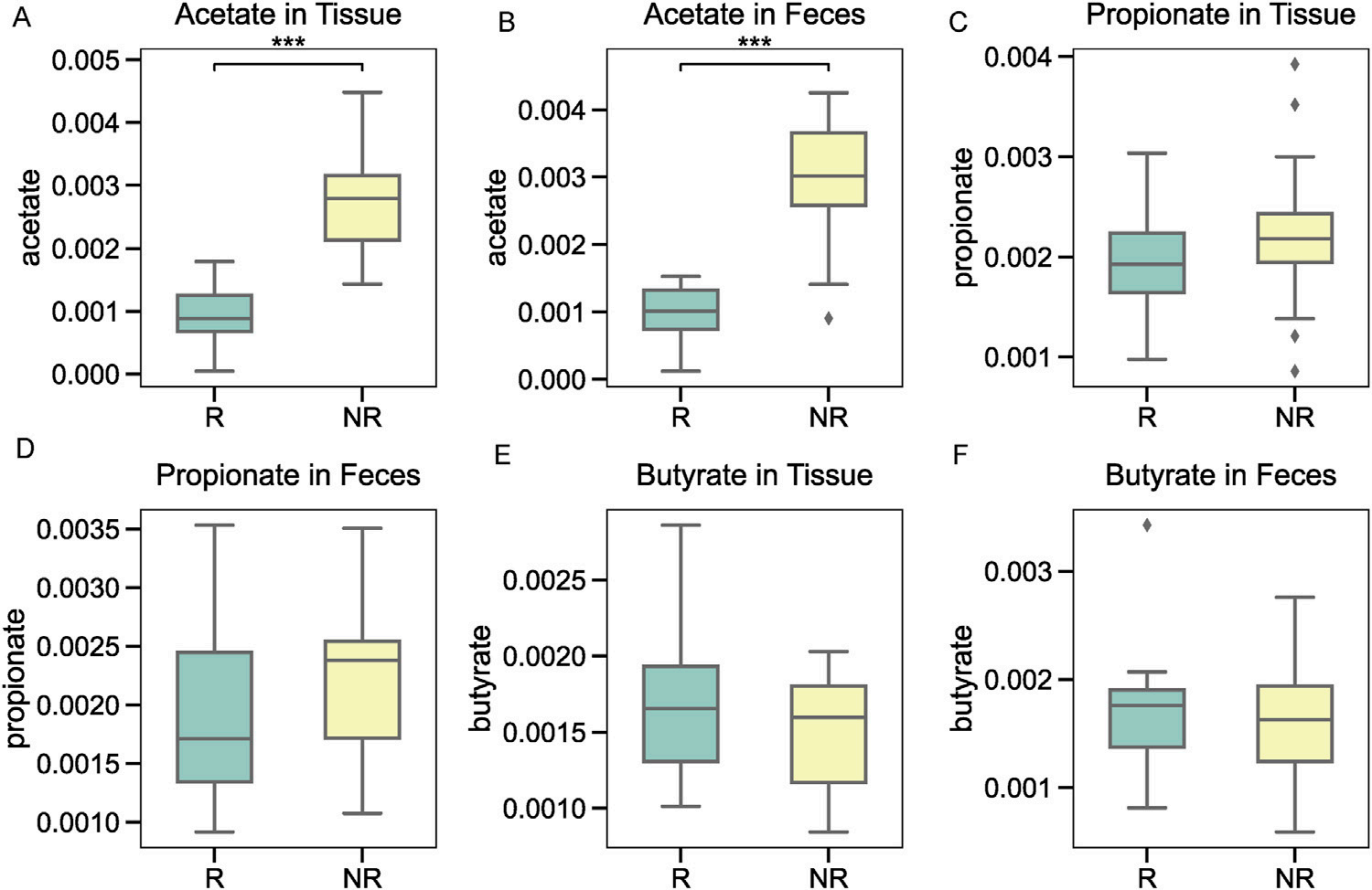
Short-chain fatty acid profiling in tumor tissue and fecal samples. **(A, B)** Acetate levels were significantly higher in the NR group in both tissue and fecal samples. **(C, D)** Propionate levels showed no significant differences between the groups. **(E, F)** Butyrate levels displayed minor variations without consistent differences between the R and NR groups.

Targeted metabolomic profiling was conducted to assess the levels of common short-chain fatty acids (SCFAs) in tumor tissue and fecal samples. Among the SCFAs analyzed, acetate levels were significantly higher in the NR group compared to the R group in both tissue (Figure 7A) and fecal samples (Figure 7B). In contrast, propionate and butyrate levels did not show consistent significant differences between the groups, with minor variations observed in tissue (Figures 7C,E) and fecal samples (Figures 7D,F). These findings highlight acetate as a potential metabolic marker linked to microbiota-mediated radiotherapy resistance in NPC patients.

## Discussion

This study provides a comprehensive analysis of the microbial and metabolomic landscapes associated with radiotherapy response in nasopharyngeal carcinoma (NPC) patients. By integrating 16S rRNA sequencing, targeted metabolomics, and machine learning approaches, we identified key microbial and metabolic markers linked to radiotherapy outcomes, shedding light on potential mechanisms underlying treatment resistance and responsiveness. The integration of these methods underscores the importance of interdisciplinary approaches in uncovering actionable biomarkers and potential therapeutic targets for clinical application.

The significant differences in alpha diversity at the species level between radiotherapy-responsive (R) and radiotherapy-non-responsive (NR) groups underscore the importance of microbial diversity in modulating radiotherapy efficacy. Previous studies have highlighted the role of the microbiota in influencing tumor microenvironment and systemic immune responses, which are critical for the success of radiotherapy (Ge et al., 2021; Marcos-Zambrano et al., 2021). Specifically, the enrichment of *Bacteroides* acidifaciens in the NR group suggests its potential involvement in creating an immunosuppressive environment (Ma et al., 2023). This bacterium has been reported to produce metabolites that can suppress T-cell activation and reduce pro-inflammatory cytokine secretion, thereby diminishing the immune response necessary for effective tumor control (Li et al., 2021). On the other hand, taxa such as Propionibacterium acnes and *Clostridium* magna, enriched in the R group, may contribute to a more favorable tumor microenvironment through mechanisms such as stimulation of antigen-presenting cells and modulation of regulatory T-cell populations (Legiawati et al., 2023). This highlights the dual role of the microbiota in either promoting or impeding tumor control, further emphasizing the need for targeted therapeutic strategies.

The application of the XGBoost machine learning algorithm allowed us to pinpoint taxa with the greatest discriminatory power between the R and NR groups (Wang et al., 2022). XGBoost was chosen over other algorithms such as Random Forest or Neural Networks due to its ability to handle small to moderate datasets efficiently and provide interpretable feature importance rankings. This rationale strengthens the methodological choices made in this study. This approach not only validated previously reported microbial associations but also uncovered novel taxa with potential roles in radiotherapy response. Notably, the genus *Acidosoma* and species such as *Propionibacterium acnes* emerged as significant predictors, emphasizing the value of machine learning in biomarker discovery (Lee et al., 2019). These findings highlight the necessity of integrating traditional microbiological methods with advanced computational tools to gain deeper insights into complex host-microbe interactions in cancer.

Metabolomic profiling further supported the microbiota findings by revealing differential levels of short-chain fatty acids (SCFAs) between the R and NR groups. Acetate, significantly enriched in the NR group across both tumor tissue and fecal samples, is known to influence tumor biology. Acetate can act as a key metabolic substrate for tumor cells, promoting histone acetylation and enhancing tumor proliferation under hypoxic conditions (Mashimo et al., 2014). Additionally, elevated acetate levels have been linked to immune evasion by promoting an immunosuppressive microenvironment via increased regulatory T-cell activity (Miller et al., 2023). Conversely, the lack of significant differences in propionate and butyrate levels suggests a more targeted role for acetate in mediating radiotherapy resistance in NPC. These findings raise important questions about the therapeutic implications of targeting acetate directly versus addressing its microbial producers, such as *Bacteroides* acidifaciens. Future research could explore the feasibility and effectiveness of these distinct strategies. These observations highlight acetate as a promising metabolic biomarker and potential therapeutic target in radiotherapy-resistant NPC.

The validation of key microbial taxa using qPCR confirmed the robustness of our sequencing and machine learning results. The consistent differential abundance of *Bacteroides acidifaciens, Propionibacterium acnes, and Clostridium magna* across tumor tissue and fecal samples highlights their potential as biomarkers for predicting radiotherapy response. The dual presence of these taxa in both tumor and fecal microbiota suggests a systemic interplay between local and distal microbial communities, which may influence radiotherapy outcomes. This interplay aligns with emerging evidence suggesting that gut microbiota can modulate systemic anti-tumor immunity, thereby impacting the efficacy of radiotherapy.

Despite the strengths of this study, several limitations must be acknowledged. First, the relatively small sample size may limit the generalizability of our findings. Future studies with larger cohorts are needed to validate these results and explore additional microbial and metabolic markers. Second, the cross-sectional nature of the study precludes causal inferences regarding the role of specific taxa or metabolites in radiotherapy response. Longitudinal studies tracking microbial and metabolic dynamics throughout radiotherapy are necessary to establish causality. Moreover, while the connection between *Bacteroides acidifaciens* and acetate is compelling, further studies are required to dissect their specific mechanistic roles and to evaluate their translational potential as therapeutic targets. Lastly, the functional implications of the identified microbial and metabolic markers remain speculative. Integrative approaches combining metagenomics, transcriptomics, and metabolomics will be crucial for elucidating the underlying mechanisms.

In conclusion, our findings underscore the critical role of the microbiota and its metabolites in shaping radiotherapy outcomes in NPC. The identification of key microbial and metabolic markers not only advances our understanding of radiotherapy resistance but also paves the way for the development of microbiota-targeted therapies to enhance treatment efficacy. By integrating multi-omics approaches and exploring both microbial and metabolic interventions, these insights hold promise for translating precision medicine into clinical practice.

## Data Availability

The original contributions presented in the study are publicly available. This data can be found here: https://ngdc.cncb.ac.cn/gsa-human/browse/HRA010448.

## References

[B1] Cullin N. Azevedo Antunes C. Straussman R. Stein-Thoeringer C. K. Elinav E. (2021). Microbiome and cancer. Cancer Cell 39 (10), 1317–1341. 10.1016/j.ccell.2021.08.006 34506740

[B2] Ge Y. Wang X. Guo Y. Yan J. Abuduwaili A. Aximujiang K. (2021). Gut microbiota influence tumor development and Alter interactions with the human immune system. J. Exp. Clin. Cancer Res. 40 (1), 42. 10.1186/s13046-021-01845-6 33494784 PMC7829621

[B3] Ghannam R. B. Techtmann S. M. (2021). Machine learning applications in microbial ecology, human microbiome studies, and environmental monitoring. Comput. Struct. Biotechnol. J. 19, 1092–1107. 10.1016/j.csbj.2021.01.028 33680353 PMC7892807

[B4] Hazrati H. Kudsk P. Ding L. Uthe H. Fomsgaard I. S. (2022). Integrated LC-MS and GC-MS-based metabolomics reveal the effects of plant competition on the rye metabolome. J. Agric. Food Chem. 70 (9), 3056–3066. 10.1021/acs.jafc.1c06306 35227064

[B5] Kiousi D. E. Kouroutzidou A. Z. Neanidis K. Karavanis E. Matthaios D. Pappa A. (2023). The role of the gut microbiome in cancer immunotherapy: current knowledge and future directions. Cancers (Basel) 15 (7), 2101. 10.3390/cancers15072101 37046762 PMC10093606

[B6] Lee Y. B. Byun E. J. Kim H. S. (2019). Potential role of the microbiome in acne: a comprehensive review. J. Clin. Med. 8 (7), 987. 10.3390/jcm8070987 31284694 PMC6678709

[B7] Legiawati L. Halim P. A. Fitriani M. Hikmahrachim H. G. Lim H. W. (2023). Microbiomes in acne vulgaris and their susceptibility to antibiotics in Indonesia: a systematic review and meta-analysis. Antibiot. (Basel) 12 (1), 145. 10.3390/antibiotics12010145 PMC9854683 36671346

[B8] Lei S. Chen L. Ji P. Li K. Li Q. Huang C. (2024). Global burdens of nasopharyngeal carcinoma in children and young adults and predictions to 2040. Oral Oncol. 155, 106891. 10.1016/j.oraloncology.2024.106891 38878356

[B9] Li K. Hao Z. Du J. Gao Y. Yang S. Zhou Y. (2021). Bacteroides thetaiotaomicron relieves colon inflammation by activating aryl hydrocarbon receptor and modulating CD4(+)T cell homeostasis. Int. Immunopharmacol. 90, 107183. 10.1016/j.intimp.2020.107183 33229197

[B10] Li Z. Zhang Y. Hong W. Wang B. Chen Y. Yang P. (2022). Gut microbiota modulate radiotherapy-associated antitumor immune responses against hepatocellular carcinoma via STING signaling. Gut Microbes 14 (1), 2119055. 10.1080/19490976.2022.2119055 36093568 PMC9467592

[B11] Lim E. S. Y. Ong Y. Chou Y. Then C. K. (2024). Interconnected influences of tumour and host microbiota on treatment response and side effects in nasopharyngeal cancer. Crit. Rev. Oncol. Hematol. 202, 104468. 10.1016/j.critrevonc.2024.104468 39103130

[B12] Liu H. Tang L. Li Y. Xie W. Zhang L. Tang H. (2024). Nasopharyngeal carcinoma: current views on the tumor microenvironment's impact on drug resistance and clinical outcomes. Mol. Cancer 23 (1), 20. 10.1186/s12943-023-01928-2 38254110 PMC10802008

[B13] Ma B. Gavzy S. J. France M. Song Y. Lwin H. W. Kensiski A. (2023). Rapid intestinal and systemic metabolic reprogramming in an immunosuppressed environment. BMC Microbiol. 23 (1), 394. 10.1186/s12866-023-03141-z 38066426 PMC10709923

[B14] Marcos-Zambrano L. J. Karaduzovic-Hadziabdic K. Loncar Turukalo T. Przymus P. Trajkovik V. Aasmets O. (2021). Applications of machine learning in human microbiome studies: a review on feature selection, biomarker identification, disease prediction and treatment. Front. Microbiol. 12, 634511. 10.3389/fmicb.2021.634511 33737920 PMC7962872

[B15] Mashimo T. Pichumani K. Vemireddy V. Hatanpaa K. J. Singh D. K. Sirasanagandla S. (2014). Acetate is a bioenergetic substrate for human glioblastoma and brain metastases. Cell 159 (7), 1603–1614. 10.1016/j.cell.2014.11.025 25525878 PMC4374602

[B16] Mattiola I. Diefenbach A. (2023). Regulation of innate immune system function by the microbiome: consequences for tumor immunity and cancer immunotherapy. Semin. Immunol. 66, 101724. 10.1016/j.smim.2023.101724 36758379

[B17] Miller K. D. O'Connor S. Pniewski K. A. Kannan T. Acosta R. Mirji G. (2023). Acetate acts as a metabolic immunomodulator by bolstering T-cell effector function and potentiating antitumor immunity in breast cancer. Nat. Cancer 4 (10), 1491–1507. 10.1038/s43018-023-00636-6 37723305 PMC10615731

[B18] Mishra S. Lin Z. Pang S. Zhang W. Bhatt P. Chen S. (2021). Recent advanced Technologies for the characterization of xenobiotic-degrading microorganisms and microbial communities. Front. Bioeng. Biotechnol. 9, 632059. 10.3389/fbioe.2021.632059 33644024 PMC7902726

[B19] Rajasekaran S. Vasudevan G. Tangavel C. Ramachandran K. Nayagam S. M. Muthurajan R. (2024). Does the gut microbiome influence disc health and disease? The interplay between dysbiosis, pathobionts, and disc inflammation: a pilot study. Spine J. 24 (10), 1952–1963. 10.1016/j.spinee.2024.06.020 38925301

[B20] Saadh M. J. Ahmed H. H. Al-Hussainy A. F. Kaur I. Kumar A. Chahar M. (2024). Bile's hidden weapon: modulating the microbiome and tumor microenvironment. Curr. Microbiol. 82 (1), 25. 10.1007/s00284-024-04004-0 39614901

[B21] Schmidt D. R. Patel R. Kirsch D. G. Lewis C. A. Vander Heiden M. G. Locasale J. W. (2021). Metabolomics in cancer research and emerging applications in clinical oncology. CA Cancer J. Clin. 71 (4), 333–358. 10.3322/caac.21670 33982817 PMC8298088

[B22] Su Z. Y. Siak P. Y. Lwin Y. Y. Cheah S. C. (2024). Epidemiology of nasopharyngeal carcinoma: current insights and future outlook. Cancer Metastasis Rev. 43 (3), 919–939. 10.1007/s10555-024-10176-9 38430391

[B23] Thapa R. Magar A. T. Shrestha J. Panth N. Idrees S. Sadaf T. (2024). Influence of gut and lung dysbiosis on lung cancer progression and their modulation as promising therapeutic targets: a comprehensive review. MedComm (2020) 5 (12), e70018. 10.1002/mco2.70018 39584048 PMC11586092

[B24] Wang R. Zhang J. Shan B. He M. Xu J. (2022). XGBoost machine learning algorithm for prediction of outcome in aneurysmal subarachnoid hemorrhage. Neuropsychiatr. Dis. Treat. 18, 659–667. 10.2147/ndt.S349956 35378822 PMC8976557

[B25] Yip P. L. You R. Chen M. Y. Chua M. L. K. (2024). Embracing personalized strategies in radiotherapy for nasopharyngeal carcinoma: beyond the conventional bounds of fields and borders. Cancers (Basel) 16 (2), 383. 10.3390/cancers16020383 38254872 PMC10814653

[B26] Yue Y. Liu Q. Liu X. Wu H. Xu M. (2021). Comparative analyses on epidemiological characteristics of dengue fever in Guangdong and Yunnan, China, 2004-2018. BMC Public Health 21 (1), 1389. 10.1186/s12889-021-11323-5 34256730 PMC8278621

[B27] Zhang R. He Y. Wei B. Lu Y. Zhang J. Zhang N. (2023). Nasopharyngeal carcinoma burden and its attributable risk factors in China: estimates and forecasts from 1990 to 2050. Int. J. Environ. Res. Public Health 20 (4), 2926. 10.3390/ijerph20042926 36833622 PMC9961544

