# Supplementary material for: Microbiota and metabolomic profiling coupled with machine learning to identify biomarkers and drug targets in nasopharyngeal carcinoma
Source: Front Pharmacol. 2025 Feb 26;16:1551411. doi: 10.3389/fphar.2025.1551411 (PMC11897916; doi:10.3389/fphar.2025.1551411)
Supplement: Supplementary file 1 [file DataSheet1.pdf]

Clinical characteristics of the 22 patients with NPC

Clinical Characteristics

Characteristic	N (%)
**Sex**	
Male	14 (63.6%)
Female	8 (36.4%)
**Age (years)**	
<55	15 (68.2%)
≥55	7 (31.8%)
**Clinical stage[1]**	
I	2 (9.1%)
Ⅱ	5 (22.7%)
Ⅲ	10 (45.5%)
IVa	4 (18.2%)
IVb	1 (4.5%)
**Tumor stage[1]**	
T1	4 (18.2%)
T2	5 (22.7%)
T3	10 (45.5%)
T4	3 (13.6%)
**Node stage[1]**	
N0	3 (13.6%)

Characteristic	N (%)
N1	6 (27.3%)
N2	10 (45.5%)
N3	3 (13.6%)
**Metastasis stage[1]**	
M0	20 (90.9%)
M1	2 (9.1%)
**Response to Radiotherapy**	
R (Response)	12 (54.5%)
NR (Non-response)	10 (45.5%)

Note: NPC, nasopharyngeal carcinoma; [1]According to the 8th edition of the UICC/AJCC staging system.
